# Ultrasound assessment of the rectus femoris in patients with chronic obstructive pulmonary disease predicts poor exercise tolerance: an exploratory study

**DOI:** 10.1186/s12890-021-01663-8

**Published:** 2021-09-25

**Authors:** Mingming Deng, Chaonan Liang, Yan Yin, Jun Shu, Xiaoming Zhou, Qiuyue Wang, Gang Hou, Chen Wang

**Affiliations:** 1 Department of Pulmonary and Critical Care Medicine, Center of Respiratory Medicine, China-Japan Friendship Hospital, Beijing, 100029 China; 2 Graduate School of Peking Union Medical College, Chinese Academy of Medical Sciences, Peking Union Medical College, Beijing, 100029 China; 3 National Center for Respiratory Medicine, Beijing, 100029 China; 4 Institute of Respiratory Medicine, Chinese Academy of Medical Sciences, Beijing, 100029 China; 5 National Clinical Research Center for Respiratory Diseases, Beijing, 100029 China; 6 Department of Pulmonary and Critical Care Medicine, First Hospital of China Medical University, Shenyang, 110001 China; 7 Institute of Clinical Medical Science, China-Japan Friendship Hospital, Beijing, 100029 China; 8 Department of Pulmonary and Critical Care Medicine, Fourth Hospital of China Medical University, Shenyang, 110001 China; 9 Chinese Academy of Medical Sciences and Peking Union Medical College, Beijing, 10029 China

**Keywords:** COPD, Rectus femoris, Ultrasound, Exercise tolerance

## Abstract

**Background:**

Reduced exercise tolerance is an important clinical feature of chronic obstructive pulmonary disease (COPD) and is associated with poor prognosis. The 6-min walk test (6MWT) is widely used to assess exercise capacity; however, it is not commonly administered in primary medical institutions because it requires a suitable site and professional training. Ultrasound has great potential for evaluating skeletal muscle dimensions in COPD. However, whether skeletal muscle ultrasound can predict impaired exercise tolerance is unclear.

**Methods:**

The study included 154 stable patients with COPD, who were randomly divided into a development set and a validation set. The thickness (RF_thick_) and cross-sectional area (RF_csa_) of the rectus femoris were measured using ultrasound. Standardized RF_thick_ (STD- RF_thick_) and Standardized RF_csa_ (STD-RFcsa) were obtained via standardization of RF_thick_ and RF_csa_ by patients' height.

**Results:**

Strong correlations were observed between the 6MWD and RF_thick_ (r = 0.84, *p* < 0.001) and between the 6MWD and RF_csa_ (r = 0.81, *p* < 0.001). In the development set, the optimal cut-off values for men and women for predicting poor exercise tolerance were < 3.098 cm/m and < 3.319 cm/m for STD-RF_thick_ and < 4.052 cm^2^/m and < 4.366 cm^2^/m for STD-RF_csa_, respectively. In the validation set, the area under the curve (AUC) values for the prediction of a 6MWD < 350 by STD-RF_thick_ and STD-RF_csa_ were 0.881 and 0.903, respectively. Finally, the predictive efficacy of STD-RF_thick_ (AUC: 0.922), STD-RF_csa_ (AUC: 0.904), and the derived nomogram model (AUC: 0.98) for exercise tolerance was superior to that of the sit-to-stand test and traditional clinical features.

**Conclusions:**

Rectus femoris ultrasound has potential clinical application to predict impaired exercise tolerance in patients with COPD.

**Supplementary Information:**

The online version contains supplementary material available at 10.1186/s12890-021-01663-8.

## Background

Chronic obstructive pulmonary disease (COPD) is the third most common cause of death globally [1]. In China, COPD is the fifth leading cause of death, with a reported prevalence of 13.7% in people aged ≥ 40 years [2, 3]. Reduced exercise tolerance is one of the main clinical features of COPD, and it is associated with increased frequency of acute exacerbations and all-cause mortality [4, 5].

The six-minute walk test (6MWT) is a common method of assessing exercise tolerance. In the 6MWT, patients walk as far as possible along a minimally trafficked 30-m corridor for a period of 6 min [6, 7]. A 6MWD < 350 m indicates impaired exercise tolerance and may predict a poor prognosis in COPD patients [8–10]. However, the 6MWT is difficult to administer in primary medical institutions because it requires an appropriate site (a 30-m flat course is required, and the layout of the track may influence the performance) [11, 12]. Establishing a screening method that is highly accurate, simple, and can be performed in primary medical facilities is therefore important.

The mechanism underlying reduced exercise tolerance in COPD patients is multifactorial [13]. Increasing evidence indicates that skeletal muscle dysfunction limit the exercise ability of patients and lead to impaired exercise tolerance [14, 15]. Identifying biomarkers related to skeletal muscle dysfunction may help predict exercise capacity in COPD patients. Recent study [16] has shown that the ultrasound assessment of the intercostal muscles can determine spirometry-related COPD severity. Ultrasound can also be used to estimate skeletal muscle dimensions of COPD patients [17, 18]. However, whether skeletal muscle ultrasound can predict impaired exercise tolerance remains unclear.

In this prospective study, we hypothesized that ultrasound of the rectus femoris could predict poor exercise tolerance in patients with COPD. Our aim was to build a nomogram based on ultrasound of the rectus femoris to predict poor exercise tolerance in patients with COPD. First, we measured skeletal muscle dimensions in COPD patients by ultrasound and analysed the correlations of the 6MWD with the thickness and cross-sectional area. In addition, we determined the cut-off values for thickness and cross-sectional area of the rectus femoris to predict impaired exercise tolerance (6MWD < 350 m). Finally, the nomogram model (combining traditional clinical features, STD-RF_thick_, and STD-RF_csa_) was found to improve the predictive ability of impaired exercise tolerance.

## Methods

### Study design and patients

A total of 154 COPD patients (≥ 40 years old) from the First Hospital of China Medical University (Shenyang, China) were recruited for this prospective observational study between August 2018 and December 2019. The inclusion criterion was a diagnosis of stable COPD according to the Global Initiative for Chronic Obstructive Lung Disease (GOLD) criteria. The exclusion criteria were as follows: COPD exacerbation within the last 1 month; presence of severe cardiovascular disease or active lung disease; concomitant disease affecting the musculoskeletal system; long-term systemic steroid therapy; and inability to read or understand the informed consent documents. Clinical features, including age, sex, height, and weight, were obtained from medical records. The study was approved by the research ethics committees of the First Hospital of China Medical University, and written informed consent was obtained from all patients.

### Pulmonary function and assessment of modified British Medical Research Council (mMRC) scale and COPD Assessment Test (CAT)

Spirometry measurements (pre-bronchodilator) were performed following the American Thoracic Society and the European Respiratory Society guidelines using a Jaeger MasterScreen system (Jaeger, Viasys Healthcare GmbH, Hoechberg, Germany). Dyspnoea symptoms were measured using the Chinese version of the mMRC dyspnoea scale [19, 20], and health status was measured using the Chinese version of the CAT [21, 22].

### Quadriceps muscle strength (QMS)

QMS was measured via a dynamometer (type: microFET2™; Hoggan, Salt Lake City, UT, USA) following the instructions in the manufacturer’s manual and previous studies [23, 24]. The knee of the patient was flexed to 90°, and the dynamometer plate was placed. The anterior end of the dynamometer was located 5 cm proximal to the lateral malleolus on the anterior surface of the leg and perpendicular to the long axis of the tibia. The participant was then instructed to generate a maximal knee extension force to hold the line in the same position for a duration of 4 s by pushing against the dynamometer plate to which the investigator applied increasing force with no encouragement (see Additional file 2: Fig. S2). The participant was then asked to generate a maximal knee extension force to hold the line in the same position for a duration of 4 s by pushing against the dynamometer plate to which the investigator applied increasing force with no encouragement. The same steps were repeated twice, with an intervening interval of 30–60 s. The average value of the last two assessments for each lower limb was recorded as the maximum unilateral contraction force. Then, the average contraction force on both sides was used to obtain the QMS.

### Fat-free mass index (FFMI)

The participant's body fat rate (BFR) was measured by a bioelectrical impedance meter (HBF-701, Omron, Japan). The FFMI was calculated as follows:

$${\text{FFMI}} = {\text{weight}}\,({\text{kg}}) \times (1 - {\text{BFR}})/{\text{height}}\,{\text{(m}})^{2} .$$

### Five-repetition sit-to-stand test (5STS) and the 30-s sit-to-stand test (30STS)

The participant was seated on a chair that measured 48 cm high and had no armrests, with their feet on the ground, back supported by the back of the chair, and hands folded in front of their chest. After hearing the test start command, the participant was asked to stand up and sit down 5 times as quickly as possible; the time needed to complete the 5 repetitions was recorded. During the test, participants were instructed to keep their arms crossed on the chest, and to completely straight the knee joint when standing. The participant was given verbal encouragement during the test. The test was performed 3 times, with 1-min rest intervals. The average of 3 tests was recorded as the result. For the 30STS, the researcher recorded the number of times the participants stood up and sat down in 30 s. The test was repeated three times, and the average value was recorded as the result.

### 6MWT

According to the 2002 American Thoracic Society (ATS) guidelines [25], a closed, long, and straight 30-m indoor corridor was selected. The test method was explained to the patient before the test, and the patient was told to walk as much as possible. If they felt short of breath or experienced chest pain or dizziness, they were allowed to slow down or to stop to rest. If the above symptoms worsened and were not relieved after rest, the test was stopped immediately, and the patient was supervised by the experimenter and encouraged using standardized language. After 6 min, the patient heard the experimenter say "time is up", which was their indication to stop. The test personnel recorded the distance travelled in metres.

### Measurements of the thickness and cross-sectional area of the rectus femoris

**Fig. 1:**
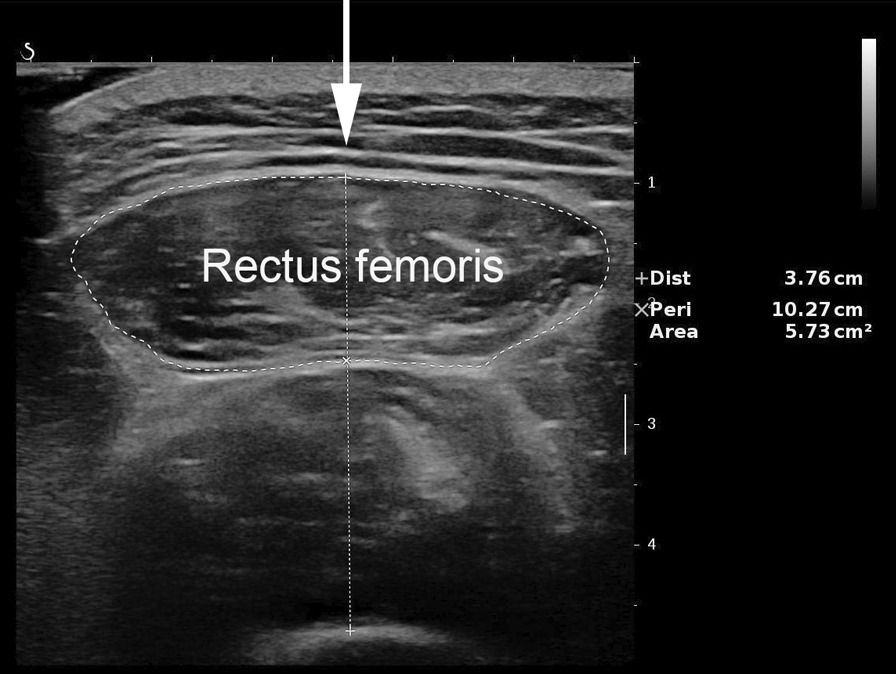
Ultrasound of the rectus femoris. The arrow indicates the direction of the scan

Measurement of the quadriceps rectus femoris thickness and cross-sectional area was performed as in previous studies [26, 27]. Greyscale ultrasound was performed with an Aixplorer ultrasound scanning system (SuperSonic Imagine, Aix-en-Provence, France) with a 4- to 15-MHz linear-array transducer. The width of linear-array transducer was 5 cm. The entire cross-sectional image of the rectus femoris in all patients were visualized via Aixplorer ultrasound scanning system. Our ultrasonographers received formal training, and with > 10 years of experience. The patient did not engage in strenuous exercise for 72 h, rested quietly for 15 min and then laid on their back on the operating bed, relaxing all their muscles. The researchers set up a bracket to fix the ultrasound probe in place, thereby reducing muscle deformation due to external forces, and placed the ultrasound probe perpendicular to the patient's dominant leg. The transducer was positioned perpendicular to the long axis of the dominant leg (precisely at 3/5 of the distance from the anterior superior iliac spine to the superior patellar border). This was the highest point in the thigh that the entire rectus femoris cross-section could be visualized in a single field in all subjects; other muscles of the quadriceps’s group could not be encompassed in this manner. The scanning depth was set such that the femur could be detected for orientation. Gentle contraction-relaxation manoeuvres were employed to delineate the muscle septa prior to image acquisition. RF_thick_ and RF_csa_ were calculated after the inner echogenic line of the rectus femoris was outlined by a movable cursor on a frozen image (Fig. 1). RF_thick_ and RF_csa_ were recorded as the averages of three consecutive measurements within 10%.

### Construction of the nomogram and decision curve analysis

A nomogram is an intuitive and effective method of displaying the results of a risk model. It was constructed using the R package “rms”. Decision curve analysis was used to decide whether the predictive nomogram was clinically useful.

### Statistical analyses

Statistical analyses were performed using SPSS 13.0 software (IBM, Armonk, NY, USA). Continuous variables are expressed as the median values and IQRs, as well as the minimum and maximum values. Spearman correlation coefficient analysis was used to compare the correlations of the RF_thick_ and RF_csa_ with the clinical features of COPD patients. The differences in the RF_thick_ and RF_csa_ between men and women were determined using unpaired t tests, and p-values < 0.05 were considered statistically significant. Receiver operating characteristic (ROC) curve analysis and the area under the curve (AUC) were used to determine the optimal cut-off values for STD-RF_thick_, STD-RF_csa_ and the STST results for the prediction of a 6MWD < 350 m.

## Results

### Patient characteristics

A total of 154 patients were enrolled in the final analysis. The baseline characteristics of the patients are listed in Table 1. The range of FEV_1_% of our patients is 30.1–110.6%. Our study population included patients of different severity, and it doesn’t limit the external validity of the study.

**Table 1:** Patient characteristics

	Overall
n	154
Age	64 (41–83)
Sex/male (%)	108 (70)
FEV_1_(L)	1.56 (0.45–3.1)
FEV_1_%pred	59.35 (30.1–110.6)
FVC (L)	2.80 (0.92–5.21)
FVC% pred	80.82 (34.4–123.8)
FEV_1_/FVC	54.90 (31–68.98)
RV (L)	4.06 (1.34–7.31)
TLC (L)	6.44 (3.52–9.84)
RV/TLC (%)	73.69 (31–242.4)
BMI (kg/m^2^)	23.92 (11.3–37.3)
FFMI (kg/m^2^)	17.00 (9.5–23.8)
Height (cm)	165.41 (141–180)
Weight (Kg)	65.07 (26.7–110.4)
mMRC	2 (0–4)
CAT	15 (0–37)
RF_thick_ (cm)	5.46 (4.5–6.97)
RF_csa_ (cm^2^)	7.05 (6.01–8.28)
6MWD (m)	369 (108–554)
QMS (kg)	42.91 (21.55–68.1)

FEV_1_, Forced Expiratory Volume in the first second**;** FEV_1_% pred, FEV percentage predicted; FVC, forced vital capacity; FVC% pred, FVC percentage predicted; RV, residual volume; TLC, total lung capacity; BMI, body mass index; FFMI, fat-free mass index; CAT, COPD Assessment Test; 6MWD, 6-min walk distance; QMS, quadriceps muscle strength

### Relationships of quadriceps thickness (RF_thick_) and quadriceps cross-sectional area (RF_csa_) with the clinical features of COPD patients

**Fig. 2:**
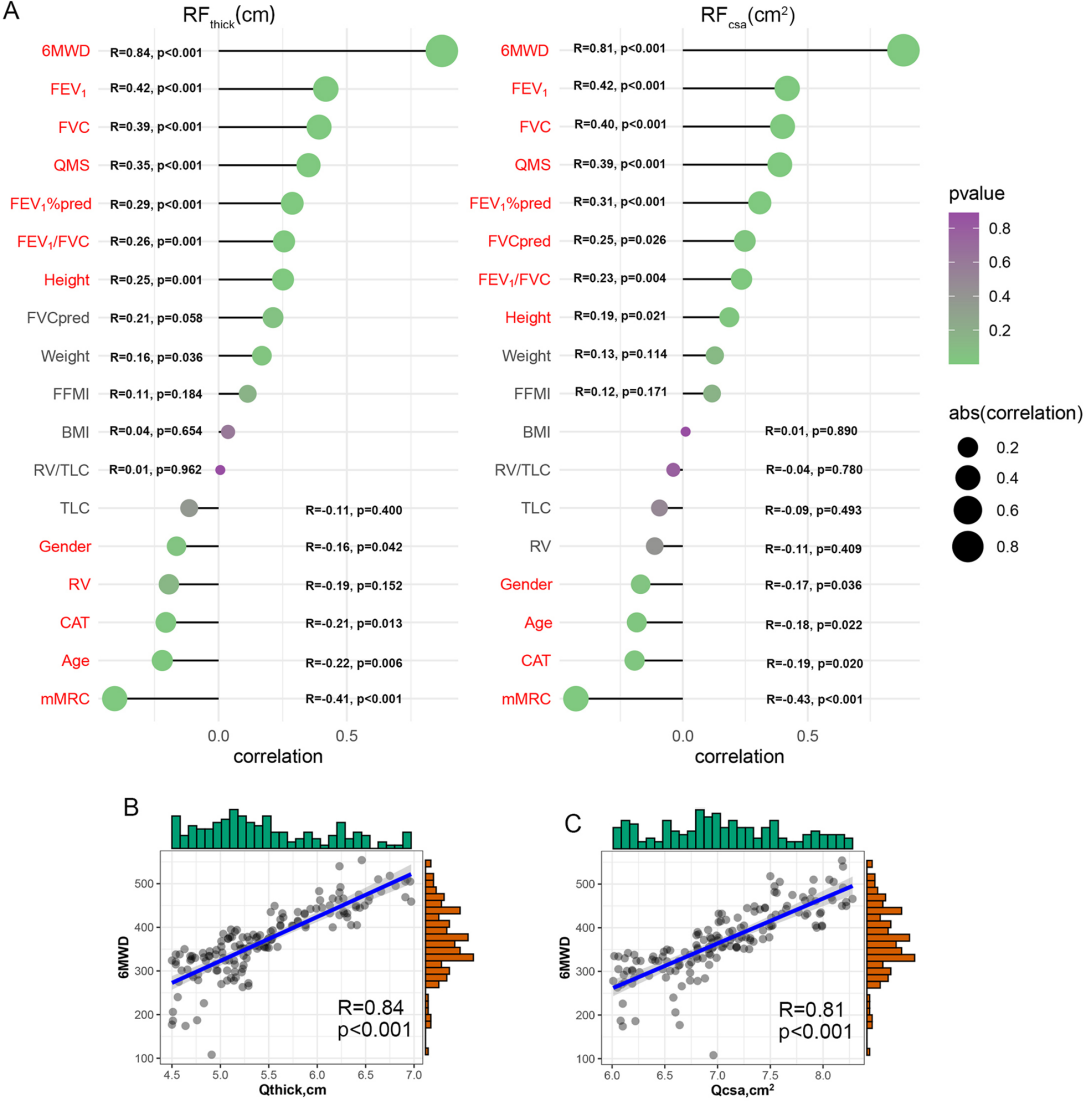
Relationships of rectus femoris thickness (RF_thick_) and rectus femoris cross-sectional area (RF_csa_) with the clinical features of COPD patients. **A**: Relationships of RF_thick_ (left) and RF_csa_ (right) with the clinical features; red: statistically significant (*p* < 0.05). The 6MWD is significantly positively correlated with RF_thick_ (**B**) and RF_csa_ (**C**). The histograms on the right and top of the figure represent the distribution of the data. The more data in this section, the higher the column

The relationships of RF_thick_ and RF_csa_ with the clinical features were analysed. As shown in Fig. 2A, the 6MWD (r = 0.870, *p* < 0.001), forced expiratory volume in one second (FEV_1_) (r = 0.418, *p* < 0.001), forced vital capacity (FVC) (r = 0.392, *p* < 0.001), QMS (r = 0.351, *p* < 0.001) and percent predicted forced expiratory volume in one second (FEV_1_%pred) (r = 0.287, *p* < 0.001) were positively correlated with RF_thick_; the CAT score (r = -0.206, *p* = 0.013) and the mMRC score (r = -0.405, *p* < 0.001) were negatively correlated with RF_thick_. The 6MWD (r = 0.883, *p* < 0.001), FEV_1_ (r = 0.418, *p* < 0.001), FVC (r = 0.399, *p* < 0.001), QMS (r = 0.388, *p* < 0.001), FEV_1_% pred (r = 0.308, *p* < 0.001), percent predicted forced vital capacity (FVC%pred) (r = 0.248, *p* = 0.026), and FEV_1_/FVC ratio (r = 0.235, *p* = 0.004) were positively correlated with RF_csa_; the CAT score (r = -0.193, *p* = 0.020) and mMRC score (r = -0.428, *p* < 0.001) were negatively correlated with RF_csa_. The 6MWD showed significant strong positive correlations with RF_thick_ (Fig. 2B) and RF_csa_ (Fig. 2C).

### Analysis and validation of STD-RF_thick_ and STD-RF_csa_ as predictors of poor exercise tolerance

**Fig. 3:**
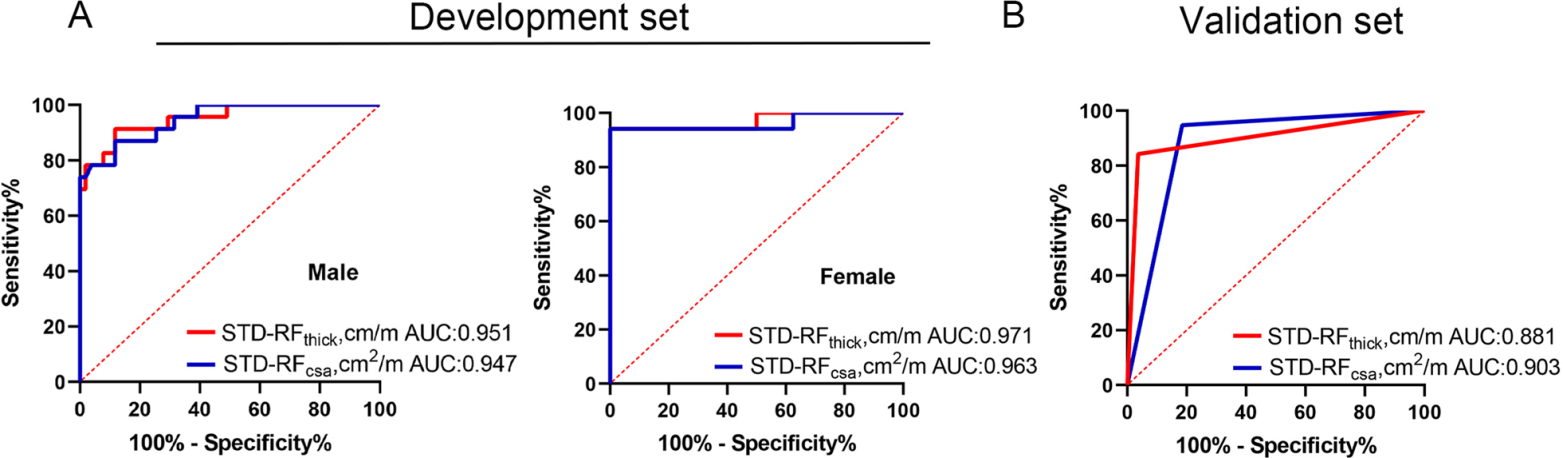
Receiver operating characteristic curve analysis of STD-RF_thick_ and STD-RF_csa_ for the prediction of poor exercise tolerance (6MWD < 350 m) in the development set (**A**) and the validation set (**B**)

A total of 154 COPD patients were randomly assigned to a development set and a validation set at a ratio of 7:3 (Table 2). Because RF_csa_ and RF_thick_ differed between men and women (Additional file 1: Fig. S1), we performed ROC analysis in men and women separately to evaluate the predictive ability of RF_thick_ and RF_csa_ for poor exercise tolerance. And, Next, we standardized RF_thick_ and RF_csa_ by patients' height, and obtained STD-RF_thick_ and STD-RF_csa_. The ROC curves derived from the development set demonstrating the ability of skeletal muscle ultrasound to predict impaired exercise tolerance based on STD-RF_thick_ and STD-RF_csa_ are shown in Fig. 3A. In male COPD patients, the sensitivity and specificity for predicting a poor 6MWD based on STD-RF_thick_ were 91.3% and 88.24%, respectively (the cut-off point was 3.098 cm/m and the AUC was 0.951), and the sensitivity and specificity for predicting a poor 6MWD based on STD-RF_csa_ were 86.96% and 88.24%, respectively (the cut-off point was 4.052 cm^2^/m and the AUC value was 0.947). In female patients, the sensitivity and specificity for predicting a poor 6MWD based on STD-RF_thick_ were 94.12% and 100%, respectively (the cut-off point was 3.319 cm/m and the AUC value was 0.971), and those for predicting a poor 6MWD based on STD-RF_csa_ were 94.12% and 100%, respectively (the cut-off point was 6.940 cm^2^/m and the AUC value was 0.963).

**Table 2:** Patient characteristics of development set and validation set

	Development set	Validation set	P value
n	108	46	
Age	64 (41–81)	65 (54–83)	0.62
Sex/male (%)	75 (69)	33 (72)	0.926
FEV_1_ (L)	1.54 (0.45–3.1)	1.63 (0.63–2.9)	0.424
FEV_1_%pred	58.12 (15.8–99.5)	62.38 (30.1–110.6)	0.263
FVC (L)	2.79 (0.98–5.21)	2.82 (0.92–4.75)	0.852
FVC% pred	80.14 (34.4–119.6)	82.72 (49.2–123.8)	0.671
FEV_1_/FVC	54.00 (31–68.59)	57.16 (32–68.98)	0.082
RV (L)	4.12 (1.97–7.31)	3.90 (1.34–6.43)	0.608
TLC (L)	6.55 (3.88–9.84)	6.14 (3.52–8.72)	0.316
RV/TLC (%)	75.58 (39–242.4)	68.53 (31–149.2)	0.522
BMI (kg/m^2^)	24.10 (11.3–37.3)	23.48 (17.2–30)	0.357
FFMI (kg/m^2^)	17.09 (9.5–23.8)	16.78 (11.8–19.3)	0.461
Height (cm)	165.42 (141–180)	165.40 (147–177)	0.991
Weight (Kg)	65.84 (26.7–110.4)	63.26 (40.2–90.3)	0.323
mMRC	2 (0–4)	2 (0–4)	0.599
CAT	15 (0–37)	14 (0–36)	0.804
RF_thick_ (cm)	5.48 (4.51–6.97)	5.41 (4.5–6.96)	0.515
RF_csa_ (cm^2^)	7.06 (6.01–8.22)	7.03 (6.06–8.28)	0.78
6MWD (m)	370.8 (108–554)	365.9 (177–510)	0.721
QMS (kg)	42.89 (21.55–68.1)	42.94 (24.2–65.05)	0.986

FEV_1_, Forced Expiratory Volume in the first second**;** FEV_1_% pred, FEV percentage predicted; FVC, forced vital capacity; FVC% pred, FVC percentage predicted; RV, residual volume; TLC, total lung capacity; BMI, body mass index; FFMI, fat-free mass index; CAT, COPD Assessment Test; 6MWD, 6-min walk distance; QMS, quadriceps muscle strength

In the validation set (Fig. 3B), the sensitivity was 94.74% and the specificity was 81.48% for detecting a 6MWD < 350 m with STD-RF_thick_ (AUC value: 0.881), whereas the sensitivity and specificity for predicting a poor 6MWD based on STD-RF_thick_ were 84.21% and 96.30%, respectively (AUC value: 0.903).

### Comparison of the predictive efficacy of skeletal muscle ultrasound and the STST

**Fig. 4:**
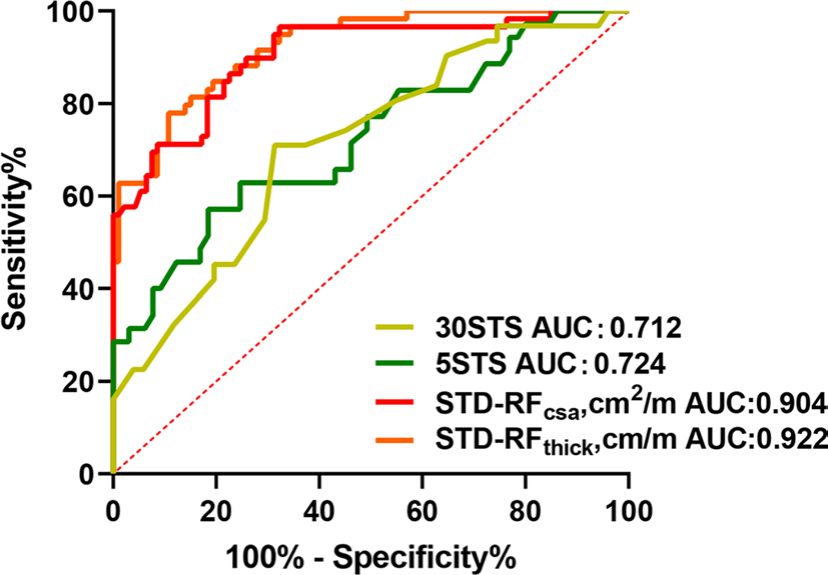
Receiver operating characteristic curve analysis of STD-RF_thick_, STD-RF_csa_, the 5STS, and the 30STS for the prediction of exercise tolerance (6MWD < 350 m)

The results from our group [24] and other studies [28–30] indicate that the STST can be used as a primary screening test to evaluate exercise tolerance in COPD patients. Therefore, we compared the efficacy of the STST to that of the STD-RF_thick_ and STD-RF_csa_ derived from skeletal muscle ultrasound in 154 COPD patients for predicting a poor 6MWD. As shown in Fig. 4, the AUC values for the 30STS score, 5STS score, STD-RF_csa_, and STD-RF_thick_ were 0.712, 0.724, 0.904, and 0.922, respectively. The sensitivities of the 30STS score, 5STS score, STD-RF_csa_, and STD-RF_thick_ were 70.97%, 82.86%, 96.61%, and 77.97%, respectively, whereas the specificities were 68.63%, 44.12%, 67.74%, and 89.25%, respectively. The above results showed that the levels of predictive power of STD-RF_thick_ and STD-RF_csa_ were higher than those of the STSTs.

### Construction of a nomogram to predict impaired exercise tolerance

**Fig. 5:**
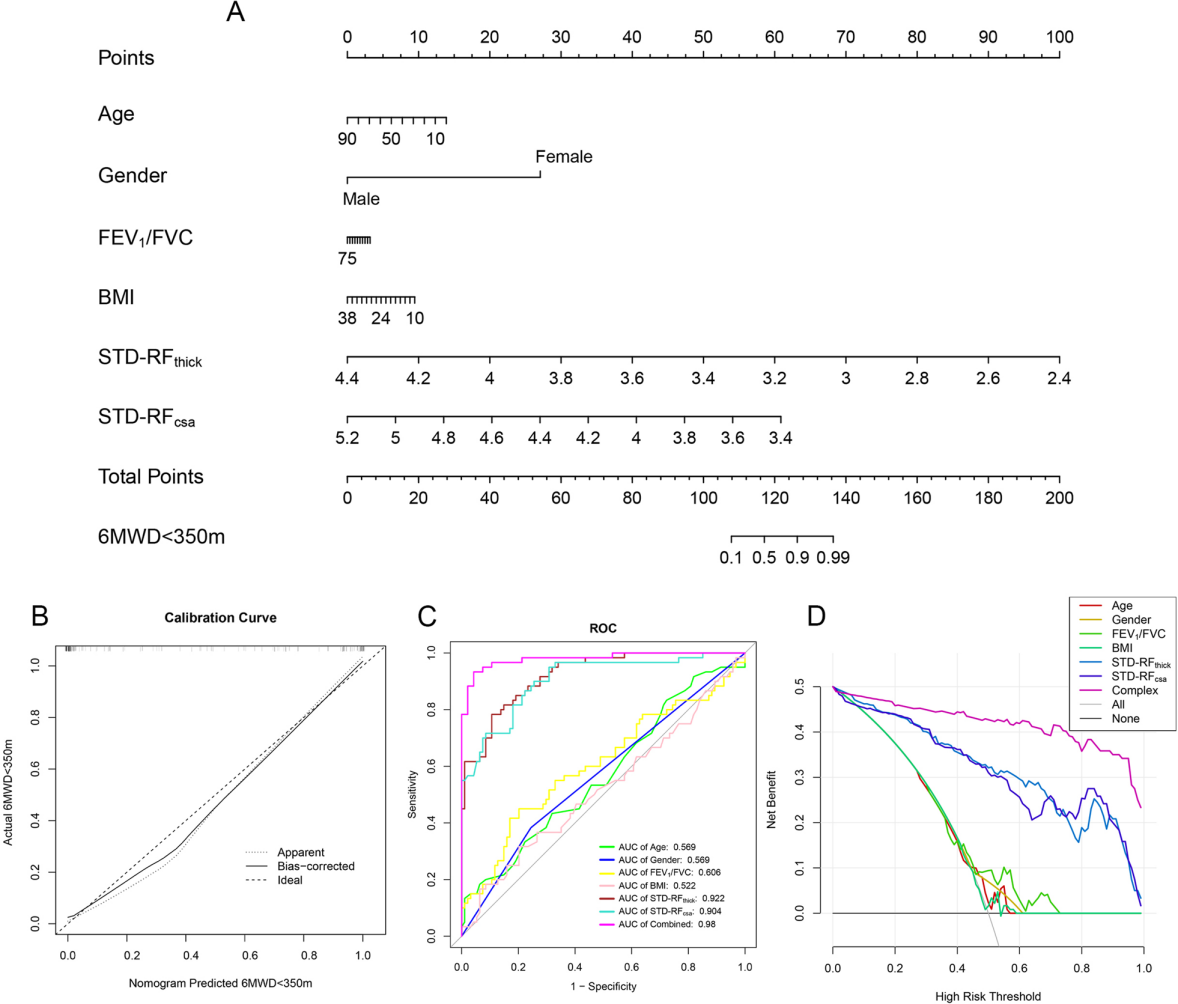
Construction of a nomogram model. **A** A nomogram was constructed to predict poor exercise tolerance (6MWD < 350 m) in COPD patients. **B** Calibration curves for the nomogram for the prediction of poor exercise tolerance (6MWD < 350 m) in COPD patients. **C** ROC curve analysis showing that the highest AUC value corresponded to the nomogram model. **D** Decision curve analysis showing the net benefit of the nomogram model for the prediction of poor exercise tolerance (6MWD < 350 m)

Exercise tolerance is affected by many factors, and has the complex mechanisms. Previous studies [31–33] have shown that Age, BMI, and pulmonary function are effective predictors of poor exercise tolerance in COPD. The Nomogram model could comprehensively incorporate the effects of diverse clinical factors. A nomogram model was constructed to predict impaired exercise tolerance (6MWD < 350 m) in COPD patients based on traditional clinical features, STD-RF_thick_ and STD-RF_csa_ (Fig. 5A). The performance of our model was confirmed via a calibration plot (Fig. 5B). Furthermore, the nomogram model had a higher AUC value than individual variables based on the results of the ROC analysis (Fig. 5C). Finally, the combined nomogram model had the highest efficacy for the prediction of a 6MWD < 350 m according to decision curve analysis (Fig. 5D). These results confirmed the potential clinical value of our nomogram model.

## Discussion

Ultrasound is a non-invasive technique that can be used to assess the physical condition of COPD patients and severity of COPD based on imaging of different areas, including the intercostal muscles and skeletal muscles [34, 35]. In this study, the thickness and cross-sectional area of the rectus femoris determined via ultrasound were correlated with the clinical features of COPD patients, including pulmonary function, clinical symptoms, and exercise tolerance. STD-RF_thick_ and STD-RF_csa_ predicted poor exercise tolerance more effectively than the STSTs. A nomogram model constructed based on RF_thick_, RF_csa_, and clinical features may help physicians with decision-making.

Skeletal muscle dysfunction, which is present in 1/3 of COPD patients and is more common in patients with severe disease, can affect both ventilatory and nonventilatory muscles, leading to a poor prognosis [36, 37]. Ultrasound is a promising tool for use in clinical practice to estimate skeletal muscle dimensions in COPD patients by measuring the thickness and cross-sectional area of skeletal muscles [17, 18]. We analysed a larger number of patients in this study than previous studies, and the results showed that RF_thick_ and RF_csa_ were related to clinical features (including pulmonary function, symptoms, and exercise tolerance) in COPD patients. These results indicated that rectus femoris ultrasound may be a useful method of assessing disease severity in COPD patients.

In this study, results showed that STD-RF_thick_ and STD-RF_csa_ had the highest AUC values, sensitivities, and specificities, suggesting that they are more valuable predictors of a poor 6MWD than the 5STS and 30STS. Furthermore, the constructed nomogram model further improved the predictive ability to identify patients with impaired exercise tolerance (AUC: 0.98) and is much more visual.

The results of our study suggest that ultrasound measurements of the rectus femoris in patients with COPD have potential clinical value in the prediction of exercise limitation. However, we must note that ultrasound measurements provide only a partial quantification of muscle dimensions. 6MWD is a well-established and validated parameter in COPD patients, that is associated with daily activity, severity of disease, and prognosis [6]. Prediction of 6MWD < 350 m through the quantification of muscle dimensions via ultrasound measurements still need more external data verification. In additional, sarcopenia, which includes both loss of muscle mass and function, has been recognized as a syndrome in patients with COPD [38, 39]. Ultrasound measurements of skeletal muscle may have potential value in the prediction of sarcopenia in these patients.

The present study has several limitations. 1, It should be considered as exploratory because participants were all from Northern China. Caution should be taken when extrapolating the clinical application of rectus femoris ultrasound to other ethnicities. 2, Most of our patients were male which reflects the higher prevalence of COPD in men (11.9%) than in women (5.4%) in China [3]. 3, Lack of a healthy control group. A previous study [18] indicates that mean RF_csa_ is reduced in patients with COPD by 25% of the mean value in healthy subjects. 4, Whether ultrasound assessment of the rectus femoris could predict exercise tolerance in healthy subjects is unclear. One study [40] conducted in older heathy men found that a greater change in muscle width corresponded to faster walking speeds, suggesting a link between muscular dimensional changes and performance during dynamic activities. Based on these previous reports, future studies are needed to determine the clinical value of ultrasound assessment of the rectus femoris in healthy subjects. 5, Lack of the data of the reproducibility of the measures for different observers is the major limitation of this study. Previous study [41] about rectus femoris ultrasound measurements in 17 men with COPD showed that differences in cross-sectional area of the rectus recorded by two experienced operators in patients with COPD were not significant. Specifically, determination coefficient (0.99) and correlation coefficients (0.998) between cross-sectional area of the rectus recorded by two experienced operators in patients with COPD were high. And, the error of measurements (0.06 ± 0.03 cm, *p* = 0.94) and percentage errors were small (1.4%). Overall, that study [41] has determined the reproducibility and reliability of measurements obtained by experienced operators. Thus, the assessment of RF_thick_ and RF_csa_ by ultrasound also necessitates training and must to be performed by treating physicians or trained radiologists. Future studies are needed to further verify the reproducibility of ultrasound and the standardized operation of ultrasound. 6, The study was limited to clinically stable patients with COPD. Whether the findings can be applied to patients with acute exacerbations of COPD and those undergoing pulmonary rehabilitation remains to be determined. These issues will be investigated in a future study.

## Conclusions

Based on the measurement of ultrasound, thickness of the rectus femoris standardized by patients' height (3.098 cm/m for men, 3.319 cm/m for women) and cross-sectional area of the rectus femoris standardized by patients' height (4.052 cm^2^/m for men, 4.366 cm^2^/m for women) have potential for the clinical assessment of exercise intolerance in patients with COPD.

## Supplementary Information

**Additional file 1: Fig. 1**. The difference of ultrasound assessment of the rectus femoris in men and women. The RF_thick_ (A) and RF_csa_ (B) differed between men and women. *P < 0.05.

**Additional file 2: Fig. 2**. The process of using the dynamometer. Measurement of quadriceps muscle strength via dynamometer.

## Data Availability

All data generated or analysed during this study are included in this published article.

## Abbreviations

COPD: Chronic obstructive pulmonary disease
6MWT: The 6-min walk test (6MWT)
RF_thick_: The thickness of the rectus femoris
RF_csa_: The cross-sectional area of the rectus femoris
STD-RF_thick_: Standardized the thickness of the rectus femoris
STD-RF_csa_: Standardized the cross-sectional area of the rectus femoris
AUC: The area under the curve
6MWD: 6-Min walk distance
mMRC: Modified British Medical Research Council
CAT: COPD Assessment Test
QMS: Quadriceps muscle strength
FFMI: Fat-free mass index
5STS: Five-repetition sit-to-stand test
30STS: The 30-s sit-to-stand test
