# Supplementary material for: Ultrasound assessment of the rectus femoris in patients with chronic obstructive pulmonary disease predicts poor exercise tolerance: an exploratory study
Source: BMC Pulm Med. 2021 Sep 25;21:304. doi: 10.1186/s12890-021-01663-8 (PMC8466975; doi:10.1186/s12890-021-01663-8)
Supplement: Supplementary file 1 — Additional file 1: Fig. 1. The difference of ultrasound assessment of the rectus femoris in men and women. The RFthick (A) and RFcsa (B) differed between men and women. *P < 0.05. [file 12890_2021_1663_MOESM1_ESM.docx]

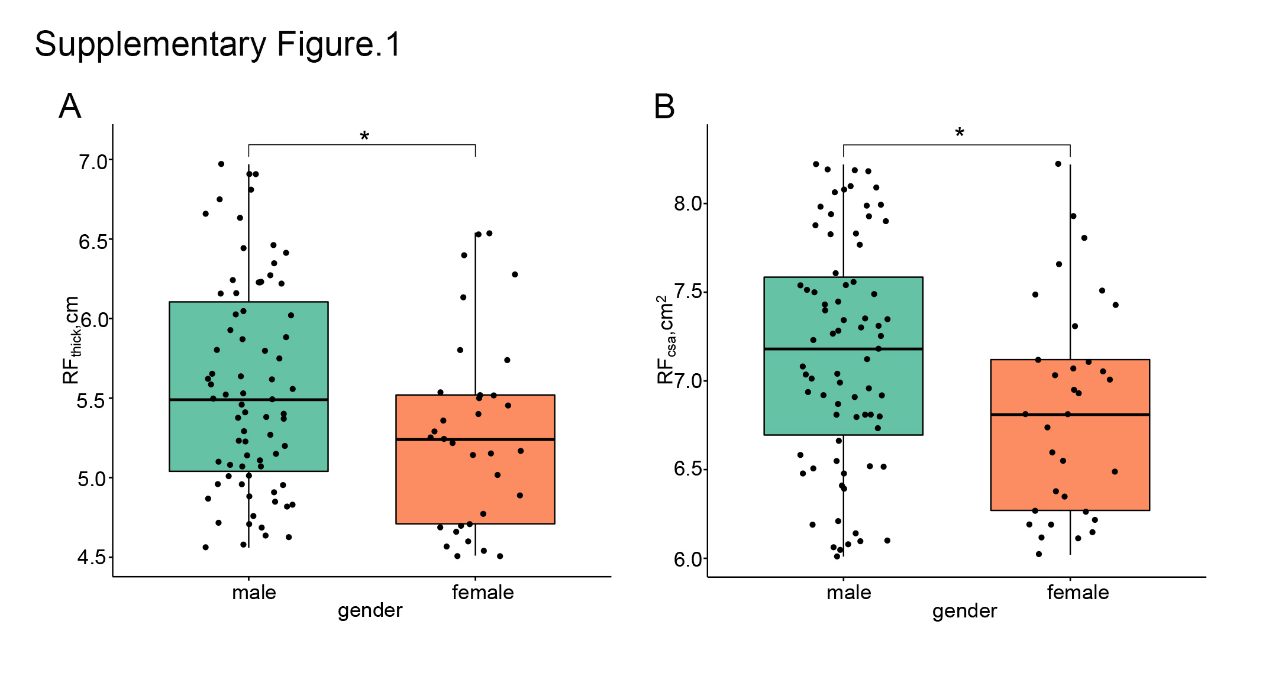
**Supplementary Figure. 1** The difference of ultrasound assessment of the rectus femoris in men and women.

The RF_thick_ (A) and RF_csa_ (B) differed between men and women. *P < 0.05.
