# Supplementary material for: Ultrasound assessment of the rectus femoris in patients with chronic obstructive pulmonary disease predicts poor exercise tolerance: an exploratory study
Source: BMC Pulm Med. 2021 Sep 25;21:304. doi: 10.1186/s12890-021-01663-8 (PMC8466975; doi:10.1186/s12890-021-01663-8)
Supplement: Supplementary file 2 — Additional file 2: Fig. 2. The process of using the dynamometer. Measurement of quadriceps muscle strength via dynamometer. [file 12890_2021_1663_MOESM2_ESM.docx]

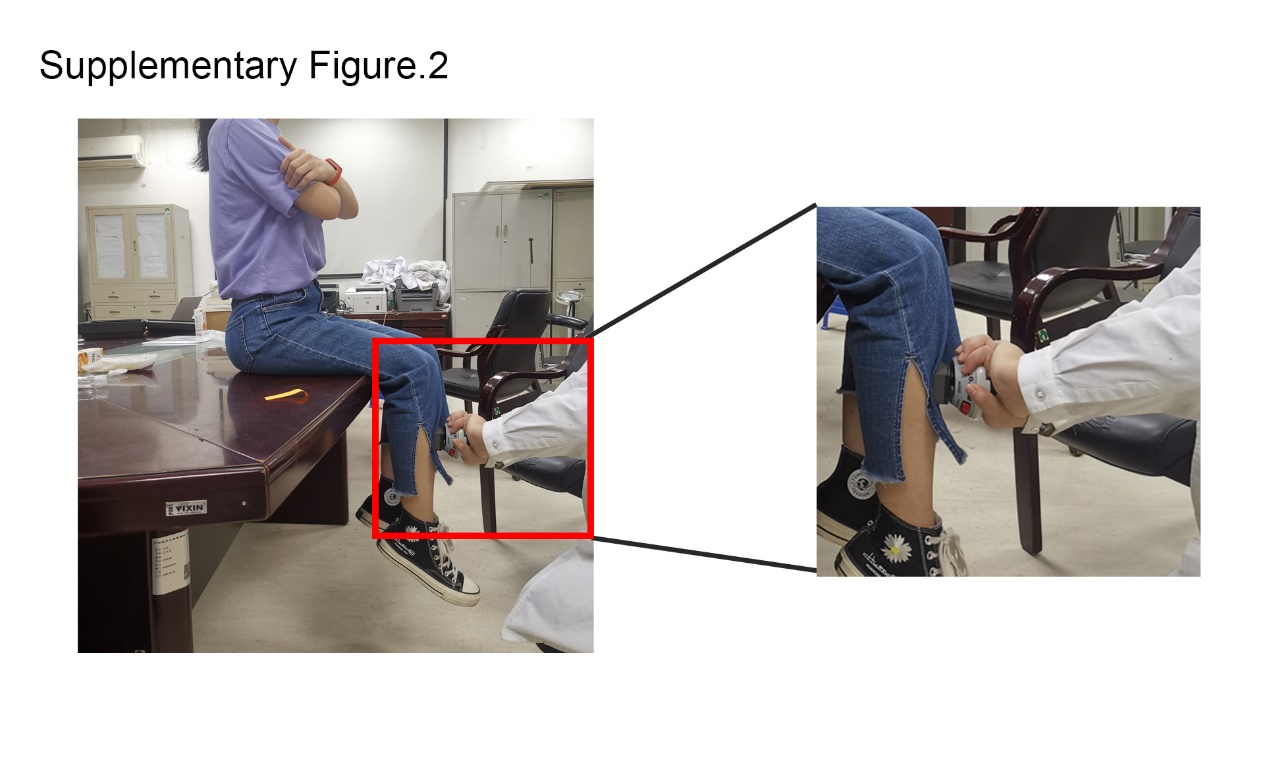
**Supplementary Figure. 2** The process of using the dynamometer.

Measurement of quadriceps muscle strength via dynamometer.
